# Diversity and evolution of plant diacylglycerol acyltransferase (DGATs) unveiled by phylogenetic, gene structure and expression analyses

**DOI:** 10.1590/1678-4685-GMB-2016-0024

**Published:** 2016-10-03

**Authors:** Andreia Carina Turchetto-Zolet, Ana Paula Christoff, Franceli Rodrigues Kulcheski, Guilherme Loss-Morais, Rogerio Margis, Marcia Margis-Pinheiro

**Affiliations:** 1 Programa de Pós-Graduação em Genética e Biologia Molecular, Departamento de Genética, Universidade Federal do Rio Grande do Sul (UFRGS), Porto Alegre, RS, Brazil.; 2 Centro de Biotecnologia e Programa de Pós-Graduação em Biologia Celular e Molecular, Universidade Federal do Rio Grande do Sul (UFRGS), Porto Alegre, RS, Brazil.; 3 Departamento de Biofísica, Universidade Federal do Rio Grande do Sul (UFRGS), Porto Alegre, RS, Brazil.; 4 Laboratório Nacional de Computação Científica, Laboratório de Bioinformática (LABINFO), Rio de Janeiro, RJ, Brazil.

**Keywords:** Triacylglycerol biosynthesis, DGAT, phylogeny, gene structure

## Abstract

Since the first diacylglycerol acyltransferase (*DGAT*) gene was characterized in plants, a number of studies have focused on understanding the role of *DGAT* activity in plant triacylglycerol (TAG) biosynthesis. *DGAT* enzyme is essential in controlling TAGs synthesis and is encoded by different genes. *DGAT*1 and *DGAT*2 are the two major types of *DGAT*s and have been well characterized in many plants. On the other hand, the *DGAT*3 and *WS/DGAT* have received less attention. In this study, we present the first general view of the presence of putative *DGAT3* and *WS*/*DGAT* in several plant species and report on the diversity and evolution of these genes and its relationships with the two main *DGAT* genes (*DGAT1* and *DGAT2*). According to our analyses *DGAT1, DGAT2, DGAT3* and *WS*/*DGAT* are very divergent genes and may have distinct origin in plants. They also present divergent expression patterns in different organs and tissues. The maintenance of several types of genes encoding DGAT enzymes in plants demonstrates the importance of DGAT activity for TAG biosynthesis. Evolutionary history studies of DGATs coupled with their expression patterns help us to decipher their functional role in plants, helping to drive future biotechnological studies.

## Introduction

Triacylglycerols (TAGs) are the major seed storage lipids, providing carbon and energy reserves to support the growth of the seedling during germination (Lisa *et al.*, 2009). TAGs are also important for pollen development and sexual reproduction in many plant species (Wolters-Arts *et al.*, 1998; Zheng *et al.*, 2003; Zhang *et al.*, 2009). These storage lipids have been intensely explored as a source of edible oils for human consumption and have also been increasingly used for non-food applications, such as fuel and industrial feedstocks (Lung and Weselake,, 2006; Durrett *et al.*, 2008; Dyer *et al.*, 2008). Oilseeds primarily accumulate five common fatty acids, namely saturated palmitic acid (C16:0), stearic acid (C18:0), unsaturated oleic acid (C18:1), and the polyunsaturated linoleic (C18:2) and α-linolenic acid (C18:3) (Millar *et al.*, 2000; Cagliari *et al.*, 2011). In addition, some plant species are able to accumulate high amounts of unusual fatty acids, such as hydroxy (*Ricinus communis*) (Li *et al.*, 2010), epoxy (*Vernonia galamensis*) (Yu *et al.*, 2008) or acetylenic fatty acids (*Euonymus alatus*) (Durrett *et al.*, 2010).

Although multiple pathways for TAG biosynthesis have been described in different organisms and tissues (Liu *et al.*, 2012), the Kennedy or sn-glycerol-3-phosphate (G3P) (Kennedy and Weiss, 1956) is the canonical pathway leading to TAG synthesis. In this pathway, the biosynthesis of TAG occurs through successive acylation reactions, which begins with the *trans* esterification of acyl-CoA to glycerol-3-phosphate to form phosphatidic acid (PA) through the action of glycerol-3-phosphate acyltransferase (G3PAT; EC 2.3.1.15) and lysophosphatidic acid acyltransferase (LPAAT; EC 2.3.1.51) enzymes. Subsequently, PA is dephosphorylated to generate diacylglycerol (DAG), which is converted to TAG through the action of acylCoA:diacylglycerol acyltransferase (DGAT; EC 3.2.1.20) (Kenedy and Weiss, 1956; Ohlrogge and Browse, 1995). Some studies about evolutionary history of Kennedy pathway enzymes were performed in the last years (Turchetto-Zolet *et al.*, 2011; Smart *et al.*, 2014, Korbes *et al.*, 2016). DGAT is considered a key enzyme in the conversion of DAG to TAG and therefore has been proposed as the rate-limiting enzyme in plant storage lipid accumulation (Ichihara *et al.*, 1988; Perry and Harwood, 1993). DGAT activity was first reported by Weiss *et al.* (1960), and in the last decade, genes encoding DGAT enzymes have been identified and studied in a variety of plant species (Hobbs *et al.*, 1999; Hobbs and Hills, 2000; He *et al.*, 2004a,b, 2006; Kroon *et al.*, 2006; Chen *et al.*, 2007; Cagliari *et al.*, 2010; Durrett *et al.*, 2010; Banilas *et al.*, 2011, Liu *et al.*, 2012). Several studies have demonstrated that DGAT plays an essential role in controlling both the quantitative and qualitative flow of fatty acids into storage TAGs (He *et al.*, 2004a; Sorensen *et al.*, 2005; Lung and Weselake, 2006). A recent study in *Brassica napus* demonstrated that suppression of the *DGAT*1 gene results in a reduction in seed oil content and germination rates, in addition to severe developmental abnormalities (Lock *et al.*, 2009). The study demonstrated that some *DGAT* genes might also have additional functions, as verified *in vitro* for *DGAT*1, which showed wax ester synthase and acyl-CoA-retinyl acyltransferase activities (Yen *et al.*, 2005). Thereby, genes encoding proteins with DGAT activity have become targets for biotechnological approaches to improve the oil content and fatty acid composition in oleaginous crops (Settlage *et al.*, 1998; Slabas *et al.*, 2001; Lung and Weselake, 2006, Lardizabal *et al.*, 2008; Xu *et al.*, 2008, Andrianov *et al.*, 2010). For example, an increase in seed oil content has been reported in *Arabidopsis thaliana* (Jako *et al.*, 2001) and *Brassica napus* (Zheng *et al.*, 2003) after *DGAT*1 overexpression. The heterologous expression of a fungal *DGAT*2 in soybean (*Glycine max*) resulted in an increase in the seed oil content (Lardizabal *et al.*, 2008). Finally, the results of forward and reverse genetic studies have also revealed that mutations in *DGAT*1 directly affect oil content in some plant species (Zou *et al.*, 1999).

DGAT enzymatic activity is encoded by different genes, which reinforces its importance in the synthesis of TAG in plants, and their distinct roles in determining the quality and quantity of acyl-CoA flux into TAG synthesis. The two major types of DGATs, designated as *DGAT1* and *DGAT2* genes, have been broadly studied in most eukaryote organisms, including fungi, animals, algae and plants. Phylogenetic and evolutionary analyses of these genes demonstrated that *DGAT*1 and *DGAT*2 evolved separately with functional convergence during eukaryotic evolution (Turchetto-Zolet *et al.*, 2011). In addition to the ubiquitous occurrence of *DGAT1* and *DGAT2* genes in plants, other DGAT-related genes have also been identified. A soluble DGAT (DGAT3) that participates on the cytosolic pathway of TAG synthesis was first identified in peanuts (*Arachis hypogaea*) (Saha *et al.*, 2006), and more recently in *A. thaliana* (Hernández *et al.*, 2012) and yeast (Rani *et al.*, 2013). In addition, a bifunctional DGAT/wax ester synthase (WS/DGAT), homologous to *Acinetobacter calcoaceticus* WS/DGAT (Kalscheuer and Steinbuchel, 2003), was characterized in *A. thaliana* (WSD1) (Li *et al.*, 2008). The *A. thaliana* WS/DGAT predominantly catalyzes the synthesis of wax esters, but it is also responsible for the synthesis of minor amounts of TAGs. While *DGAT*1 and *DGAT*2 have been well characterized in most plant species, *DGAT*3 and *WS/DGAT* were studied in very few species. Until now, little is known about the roles of *DGAT*3 and *WS/DGAT* genes in most plant species. Hence, some issues such as (i) the presence of the homologous to *DGAT*3 and *WS/DGAT* genes in other plant species, (ii) the origin of these genes, and (iii) its relationships with *DGAT1* and *DGAT2* genes, remain unsolved. Therefore, the identification of putative *DGAT3* and *WS*/*DGAT* genes and the understanding of their evolutionary history in plant species represent an important step to fully explore the DGAT potential in oilseed metabolic engineering and biotechnology.

Here, using homology searches in several plant genomes available we identified putative *DGAT3* and *WS/DGAT* genes and used a phylogenetic approach and gene structure comparison to report on the diversity and evolution of these putative *DGAT* genes. The relationship of *DGAT3* and *WS*/*DGAT* with the two main *DGAT* genes (*DGAT1* and *DGAT2*) was also discussed. In addition, aiming to the understanding of the role of these genes in an oleaginous plant during the accumulation of lipid reserves in developing seeds we evaluated the expression profile of the putative *DGAT3* and *WS/DGAT* genes in soybean (*Glycine max*). This oilseed species is one of the most economically important oilseed crops worldwide (Dornbos Jr and Muller, 1992; Saski *et al.*, 2005; Vijav *et al.*, 2009), which makes this species a potential biofuel feedstock (Hausman 2012; Hou *et al.*, 2011). The combination of experimental and *in silico* analyses allowed us to describe the molecular evolution of these DGAT genes and to infer about their possible functions. We found that like *DGAT1* and *DGAT2* genes, *DGAT*3 and *WS/DGAT* also have experienced a distinct evolutionary history with different origins. Combined, our findings improve the current understanding about plant TAG biosynthesis, and will guide future functional and biotechnological studies.

## Materials and Methods

### Data sources and sequence retrieving

*DGAT3* and *WS*/*DGAT* genes and proteins sequences were obtained through BLAST searches (TBLASTX, BLASTX and BLASTP) of the protein and genome databases with the default parameters and an e-value threshold of 1.0 e^-20^ at the NCBI (National Center for Biotechnology Information), and the completed genome projects at the Phytozome database. The DGAT3 and WSD1 sequences from *Arabidopsis thaliana* were used as queries in the BLAST searches. Supplementary Table S1 provides a detailed description of the sequences used in this study and their corresponding accession numbers. Taxa terminologies are abbreviated using the first letter of the genus and two letters of the species name (e.g., Gma corresponds to *Glycine max*).

### Sequence alignment and phylogenetic analysis

The nucleotide and protein sequences were aligned using MUSCLE (Edgar 2004) implemented in Molecular Evolutionary Genetics Analysis (MEGA version 5.0; Tamura *et al.*, 2011). The multiple alignments were manually inspected and edited and only unambiguously aligned positions were included in the final analysis. The phylogenetic analysis was constructed after protein sequence alignments using Bayesian method, carried out in BEAST1.7 software (Drummond and Rambaut, 2007). The model of protein evolution used in this analysis was the JTT model for protein matrix substitution. The Yule tree was selected as a tree prior to Bayesian analysis and 20,000,000 generations were performed with Markov chain Monte Carlo (MCMC) algorithms. The trees were visualized and edited using FigTree v1.3.1 software.

### Gene and protein structure analyses

The structural organization of the putative DGAT3 and WS/DGAT genes was determined by analyzing the genomic and coding sequences. We use the GSDraw web server, an interface for gene structure annotation available in PIECE database (Wang *et al.*, 2012). Basically, we submitted a query sequence set (in multi-FASTA format) consisting of genomic and CDS to GSDraw and retrieved the gene structures with conserved protein motifs and phylogenetic trees. In addition, we searched for predicted transmembrane structures using the transmembrane prediction server TMHMM-2.0 and SMART database with the complete putative protein sequences.

### Plant material, RNA extraction and cDNA preparation

Soybean leaf tissue (*Glycine max* cv. Conquista) and four seed developmental stages, representing R-stages (Supplementary Figure S1) (R5: beginning seed; R6: full seed; R7: beginning maturity and R8: full maturity) were collected (Egli, 1994; Egli and Bruening, 2000). Total RNA was extracted using Trizol (Invitrogen), and the RNA quality was evaluated by electrophoresis on a 1.0% agarose gel. The reverse transcription of first-strand cDNA was performed with 2 μg of purified mRNA, T25V primer (1 μg/μL) and 200 units of M-MLV reverse transcriptase (Promega) in a final volume of 50 μL. The reverse transcription reaction included a denaturation step at 70 °C for 5 min, followed by a rapid thaw on ice, and an elongation step at 42 °C for 1 h. The cDNA products were diluted 1:10 and stored at -80 °C.

### RT-qPCR expression analysis of putative soybean *DGAT3* and *WS/DGAT* genes

To analyze expression pattern of the putative *DGAT3* and *WS*/*DGAT* genes in soybean tissues, comparing with *DGAT1* and *DGAT2* expression, quantitative real time PCR (RT-qPCR) was performed using the CFX384 Real Time PCR system (BioRad) with SYBR-Green according to the manufacturer's protocol. Briefly, 10 μL of 1:100 diluted cDNA was mixed with primer pairs (0.2 μM), dNTPs (25 μM), 1X reaction buffer, MgCl_2_ (3 mM), 0.1X SYBR-Green Platinum *Taq* polymerase (0.25 U/μL) and DNase-free water to a final reaction volume of 20 μL. The RT-qPCR conditions were: an initial hot-start step at 94 °C for 5 min followed by 40 cycles of denaturation at 94 °C for 15 s, annealing at 60 °C for 10 s, extension at 72 °C for 15 s and an additional data recording step at 60 °C for 35 s. After cycling, an additional melting curve step was performed.

The four protein-coding genes, *ELF1B, CYP2, ACT* and *TUA* were selected based on previous reports as reference genes for soybean (Jian *et al.*, 2008; Hu *et al.*, 2009; Kulcheski *et al.*, 2010). The primers used in these experiments are listed in Table S2. The experiments were performed using biological and technical quadruplicates. The relative expression of the *DGAT* genes was calculated using the 2^-ΔΔCt^ method (Livak and Schmittgen, 2001). The statistical analyses were performed with SPSS v.20. One-way ANOVA was applied, with the Tukey's test (p ≤ 0.05) to compare pairwise differences in the expression for all genes.

### *In silico* expression analysis

Tissue specificity and intensity of expression of *DGAT* genes were examined using microarray data at the GENEVESTIGATOR web site (Hruz *et al.*, 2008). The available Hierarchical Clustering tool was used to perform this analysis. The highest expression values were considered for genes with more than one probe set. The expression data were gene-wise normalized and hierarchically clustered based on Pearson's coefficients.

## Results

### Homology search for putative *DGAT3* and *WS/DGAT* genes in plant genomes

Putative homologs of the *DGAT3* and *WS/DGAT* genes were searched in fully sequenced genomes from 20 plant and two algae from the Phytozome database using TBLASTX, BLASTX and BLASTP (see Material and Methods). Using *DGAT3* and *WSD1* (*WS*/*DGAT*) from *A. thaliana* as queries in blast searches, we were able to identify putative *DGAT3* and *WS*/*DGAT* homologous sequences in all genomes. The exception was the green algae species *Volvox carteri,* that present putative *DGAT3* gene but no match to *WS*/*DGAT* gene. The complete list of genes and species studied are summarized in Table S1. In total, we identified 25 putative *DGAT3* and 80 putative *WS/DGAT* genes in plant and algae genomes (Table 1). While one or two putative *DGAT3* genes were identified in all species, a larger number of putative *WS*/*DGAT* genes were found in the majority of plant species. The species included in phylogenetic and exon-intron comparisons analyses are indicated in Table 1.

**Table 1:** Number of putative *DGAT3* and *WS*/*DGAT* sequences retrieved in this study and identification of species used for the phylogenetic and exon-intron structure comparative analyses.

Species name	Taxa terminologies	N of putative *DGAT3* genes	N of putative *WS*/*DGAT* genes
*Arabidopsis thaliana*	Ath* # Δ	1	11
*Arabidopsis lyrata*	Aly*	1	7
*Brassica rapa*	Bra* # Δ	1	12
*Gossypium raimondii*	Gra*	1	5
*Theobroma cacao*	Tca	1	2
*Ricinus communis*	Rco* # Δ	1	3
*Manihot esculenta*	Mes* Δ	2	4
*Populus trichocarpa*	Ptr* Δ	1	4
*Medicago truncatula*	Mtr* # Δ	1	1
*Glycine max*	Gma* # Δ	2	1
*Solanum tuberosum*	Stu*	1	4
*Solanum lycopersicum*	Sly* Δ	1	4
*Aquilegia coerulea*	Aco* Δ	1	1
*Sorghum bicolor*	Sbi* Δ	1	3
*Oryza sativa*	Osa* # Δ	1	3
*Setaria italica*	Sit*	1	5
*Zea mays*	Zma* # Δ	1	1
*Brachypodium distachyon*	Bdi*	1	5
*Selaginella moellendorfii*	Smo* # Δ	1	2
*Physcomitrella patens*	Ppa* # Δ	2	1
*Volvox carteri*	Vca* # Δ	1	–
*Ostreococus lucimarinus*	Olu*	1	1

* Species used to perform the phylogenetic analysis shown in Figure S1

# Species used to perform the phylogenetic analysis shown in Figure 1

Δ Species used to perform the exon-intron comparisons (Figures 2, 3 and 4).

### Phylogenetic relationship of *DGAT* genes in plants

**Figure 1:**
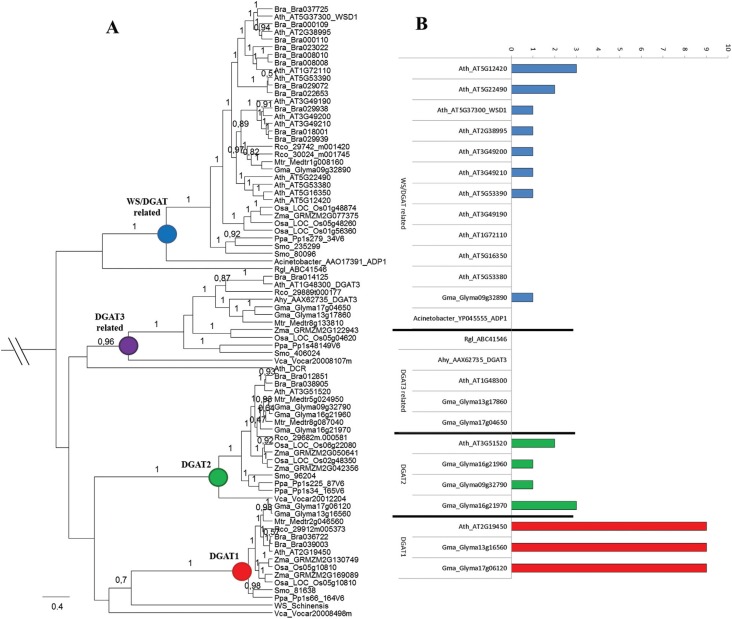
Phylogenetic relationship among plant DGAT1, DGAT2, DGAT3 and WS/DGAT protein sequences. (A) The phylogenetic analysis was performed with DGAT protein sequences from *Glycine max* (Gma), *Arabidopsis thaliana* (Ath), *Brassica rapa* (Bra), *Ricinus communis* (Rco), *Medicago truncatula* (Mtr), *Arachis hypogaea* (Ahy), *Oryza sativa* (Osa), *Zea mays* (Zma), *Selaginella moellendorffii* (Smo)*, Physcomitrella patens* and *Volvox carteri* (Vca). The DCR (AT5G23940) from *A. thaliana*, WS (AAD38041) from *Simmondsia chinensis*, the DGAT3 (ABC41546) from *Rhodotorula glutinis* and WS/DGAT (YP045555) from *Acinetobacter* sp. were also included in the analysis. The posteriori probabilities are labeled above the branches. Only values higher than 0.5 are presented. (B) Predicted transmembrane domain for DGAT1, DGAT2, DGAT3 and WS/DGAT from *A. thaliana* (Ath) and *G. max* (Gma). The WS/DGAT from *Acinetobacter* sp. and DGAT3 from *R. glutinis* and *A. hypogaea* were also analyzed. TMHMM web tools of the Center for Biological Sequence Analysis, Technical University of Denmark TMHMM Server plots showing the probability of the ALDH sequence forming a transmembrane helix (0-1.0 on the y-axis) (shown in red for the relevant amino acid sequences).

To understand the evolutionary relationships of the four different DGAT types in plant and algae species, we conducted a phylogenetic analysis using the protein sequence of putative DGAT3 and WS/DGAT identified by homology search and the DGAT1 and DGAT2 protein sequences reported in Turchetto-Zolet *et al.* (2011) (Figure 1A). For this analysis, we used DGATs sequences from nine plant and one algae species. We also included DGAT3 sequence from *Arachis hypogaea*, DCR sequence from *A. thaliana*, Wax ester synthase (WS) from *Simmondsia chinensis*, DGAT3 from *Rhodotorula glutinis* and WS/DGAT from *Acinetobacter* sp. A total of 80 sequences and 253 positions were included in the final dataset. We also performed a phylogenetic analysis of DGAT3 and WS/DGAT including a larger number of plants (Figure S2). For this analysis, we used DGAT3 and WS/DGAT sequences from 21 plant and one algae species. A total of 105 sequences and 247 positions were included in the final dataset. The phylogenetic analysis of the DGATs amino acid sequences resulted in a well-resolved tree, revealing the formation of four well-supported clades separating the different DGAT types (Figure 1A and Figure S2). Within each clade, we also observed that monocots and eudicots form distinct clusters, as was previously observed for *DGAT1* and *DGAT2* genes (Turchetto-Zolet *et al.*, 2011). The ADP1 (WS/DGAT) sequence from *Acinetobacter* sp. grouped within the WS/DGAT clade, together with *A. thaliana* WS/DGAT and putative WS/DGAT from other plant and algae species, with high support, suggesting that diversification of this DGAT type occurred before the origin of plants. The DCR (Defective Cuticle Ridge) from *A. thaliana*, which is a soluble protein that belongs to the BAHD family of acyltransferases, was related to soluble DGAT3 clade. The Wax synthase (WS) sequence, which catalyzes the final step in the synthesis of linear esters (waxes) in *Simmondsia chinensis,* is closely related with DGAT1 sequences, suggesting a common origin for DGAT1 and WS/DGAT (Figure 1A). Within DGAT3 clade, the two putative soybean *DGAT3* genes grouped closest to the *DGAT3* from peanut. Another interesting result observed in Figure 1A and Figure S2 was the gene duplication during *DGATs* gene family evolution. The pattern of gene duplication was distinct within each *DGAT1, DGAT2, DGAT3* and *WS*/*DGAT*. While *WS*/*DGAT* was the most diversified gene with all plants presenting more than two *WS*/*DGATs, DGAT3* genes was maintained as single copy in plants, except for *G. max* that has suffered gene duplication (Figure 1A and Figure S2). In *DGAT1* and *DGAT2* more than one gene was observed in most analyzed plants (Figure 1A). All duplication events seemed to have occurred after plant diversification, since one gene of each *DGAT1, DGAT2, DGAT3* and *WS*/*DGAT* was identified in algae.

The transmembrane (TrM) domains were predicted and compared among the four *DGAT* types in soybean and *A. thaliana* species (Figure 1B). This analysis demonstrated distinct structure pattern among the different DGAT types within these two species. *A. thaliana* and soybean DGAT1 proteins contain nine putative transmembrane domains (TrM) (Figure 1B). DGAT2 from soybean and *A. thaliana* have two to three TrM and the WSD1 from *A. thaliana* and the putative soybean homologous contained one TrM. In contrast, no TrM regions were detected in DGAT3, supporting their status of soluble enzymes (Figure 1B). Interestingly, some *A. thaliana* WSD1 homologous presented two and three TrM regions, while others presented no TrM regions.

### Structural organization of *DGAT3* and *WS/DGAT* genes in plants

**Figure 2:**
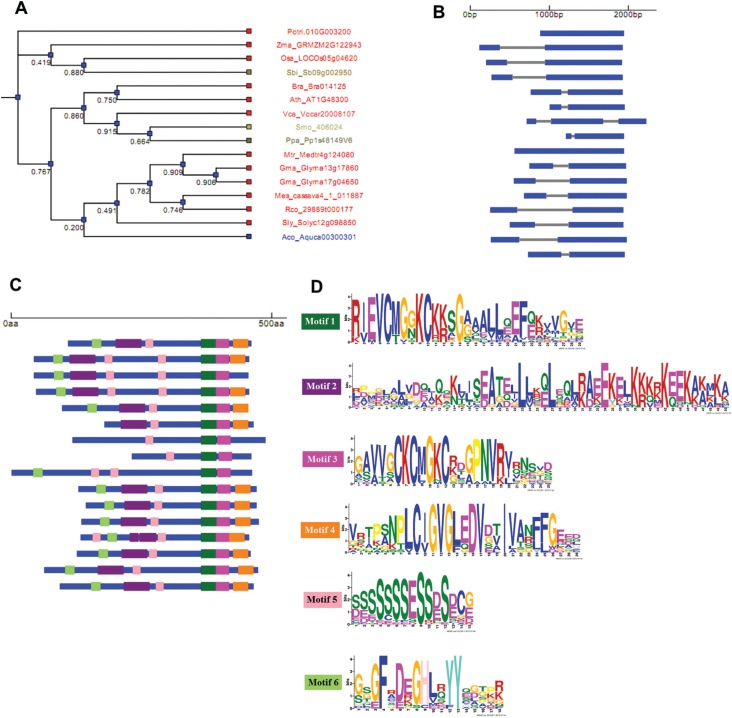
DGAT3 gene structure and organization in plant genomes. Dendrogram of sequences clustered according to the presence and similarity of identified protein motifs (A). Diagram displaying information of the gene structure for each sequence (B). Conserved motifs identified on protein sequences (C) and sequence logo of the conserved motif (D). Exon sequences are represented as blue boxes and the gray bars represent introns. The species included in this analysis are listed in the Table 1. The bootstrap values are given below the branches of the tree.

**Figure 3:**
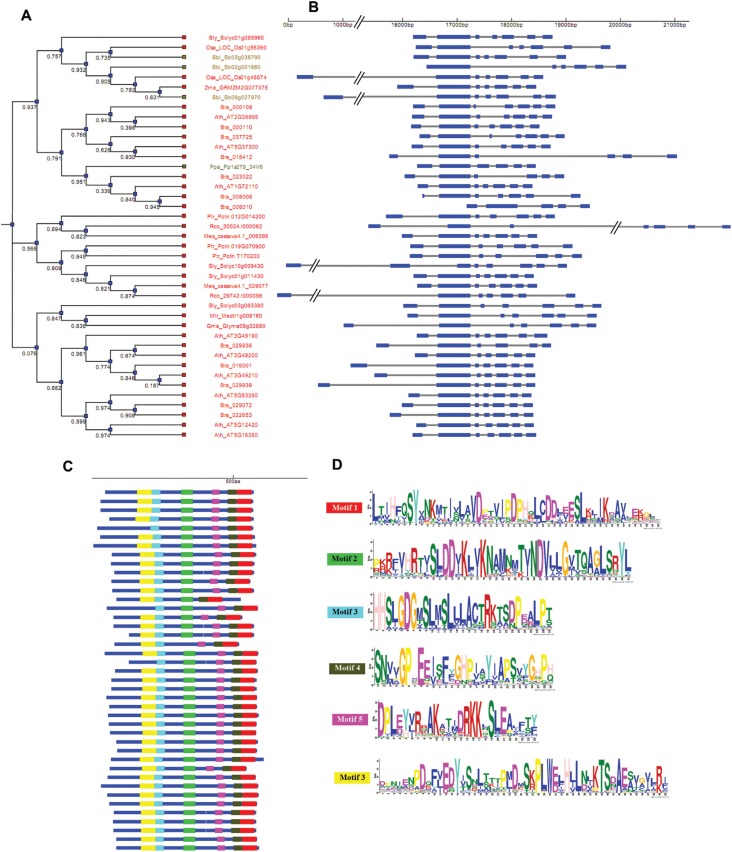
WS/*DGAT* gene structure and organization and conserved motifs identified in plant genomes. Dendrogram of sequences clustered according to the presence and similarity of identified protein motifs (A). Diagram displaying information of the gene structure for each sequence (B). Conserved motifs identified on protein sequences (C) and sequence logo of the conserved motif (D). The species included in this analysis are listed in the Table 1. Exon sequences are represented as blue boxes and the gray bars represent introns. The bootstrap values are given below the branches of the tree.

We performed a comparative analysis of the exon-intron organization of *DGAT3* and *WS/DGAT* genes in plants and algae genomes to unveil their structural organization and to infer about their molecular evolution. For this analysis, genes from 14 species of plant and one species of algae were used (Table 1). The gene structure and conserved protein motif pattern diagram linked to a bootstrapped similarity dendrogram was obtained (Figure 2A-D and Figure 3A-D). The putative *DGAT3* genes present in most species of this study contain two exons (Figure 2B). Exceptions were the green algae *V. carteri* that presents three exons, and the moss *Physcomitrella patens* and the tree species *Populus trichocarpa* that lack introns, suggesting the occurrence of gain and loss of introns during plant evolution. This analysis revealed a high degree of conservation among species regarding their gene structure, as shown in the cladogram of Figure 2B. The well characterized *DGAT3* from *A. thaliana* presents two exons. Figure 2C shows the conserved motifs identified in the protein sequences of all putative DGAT3 analyzed. We observed that these protein motifs are present in most species with a high degree of conservation. The sequences of the six domains identified are showed in Figure 2D and in the alignment of Figure S3.

The putative *WS/DGAT* genes from most species present seven exons, which were also observed in the well characterized Arabidopsis *WDS1* gene. The *WDS1* and putative *WS/DGAT* genes identified in our study are highly conserved in terms of their structural organization in all species (Figure 3 A-D). This conservation is observed even among those genes that present six and eight exons (Figure 3B), which may be related to exon loss or gain during evolution. Concerning the distribution of protein motifs in the WS/DGAT protein sequences, we observed that the six identified domains are highly conserved in most species (Figure 3C). Likewise the *A. calcoaceticus* WS/DGAT and the *A. thaliana* WSD1 protein sequences, we observed the presence of the proposed active-site motif (^228^HHXXXDG^234^) in the N-terminal region in all putative WS/DGAT identified (Figure 3C, D and Figure S4).

Comparison of the structural organization among the four different types of *DGAT* (*DGAT1, DGAT2, DGAT3* and *WS*/*DGAT*) genes from soybean and Arabidopsis revealed distinct degrees of conservation in gene structure among these genes (Figure S5). However, the comparisons clearly demonstrate a high degree of conservation within each type of *DGAT* gene between soybean and *A. thaliana* species. The *A. thaliana* and soybean *DGAT1* genes contained 16 exons, the *DGAT2* genes contained 5 to 9 exons, *DGAT3* contained 2 and *WS*/*DGAT* contained 7 exons. This demonstrates that the four types of *DGAT* genes have experienced different evolutionary history.

### Expression profiles of soybean *DGAT3* and *WS*/*DGAT*

**Figure 4:**
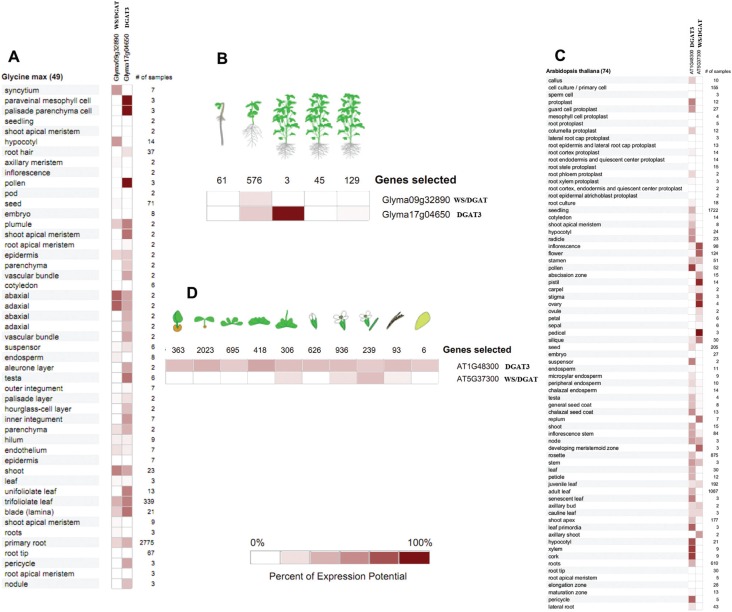
*In silico* expression analysis of the selected *DGAT* genes in soybean and *Arabidopsis*. The soybean and Arabidopsis *DGAT* genes were analyzed for organ-specific and developmental expression patterns using the GENEVESTIGATOR program. Expression profile of organ-specific (A) and plant developmental expression profiles (B) of soybean *DGAT3* and *WS*/*DGAT* genes. Expression profile of organ-specific (C) and plant developmental expression profiles (D) of Arabidopsis *DGAT3* and *WS*/*DGAT* genes.

For inference on a role of the *DGAT3* and *WS/DGAT* genes in lipid accumulation during seed development, we performed an expression analyses of the putative soybean *DGAT3* and *WS/DGAT* genes and compared the results with the microarray expression data publicly available for *A. thaliana*. First, we performed an *in silico* comparative gene expression analysis with soybean and Arabidopsis *DGAT3* and *WS/DGAT* genes using the GENEVESTIGATOR web-based software (Figure 4A). The probe sets used for *in silico* expression analysis are shown in Table S3. The analysis of the microarray expression data showed that the soybean and *A. thaliana DGAT3* and *WS/DGAT* genes present different expression patterns across different tissues and plant developmental stages within each species (Figure 4). DGAT3 transcripts of soybean were detected in 30 of 49 analyzed tissues and in three of five plant development stages, while WS/DGAT transcripts were detected in 20 of 49 tissues and in one of five analyzed plant development stages (Figure 4A,B). The same pattern was observed for *A. thaliana*, where DGAT3 transcripts were detected in 47 of 74 tissues and in all analyzed plant development stages, while WS/DGAT transcripts were detected in 23 of 74 tissues and four of 10 plant development stages (Figure 4C, D). The putative soybean *DGAT3* gene was highly expressed in paraveinal mesophyll cells, palisade parenchyma cells, pollen, plumule of the seed, shoot apical meristem, testa, unifoliolate and trifoliolate leaves, while the putative soybean *WS/DGAT* was highly detected in syncytium, hypocotyl, adaxial and abaxial cotyledon (Figure 4A,B). In *A. thaliana, DGAT3* was more expressed in radicle, pollen, senescent leaf, leaf primordia, xylem and cork, while *WS/DGAT* was higher in inflorescence, flower, pistil, stigma, ovary and pedicel (Figure 4C,D).

**Figure 5:**
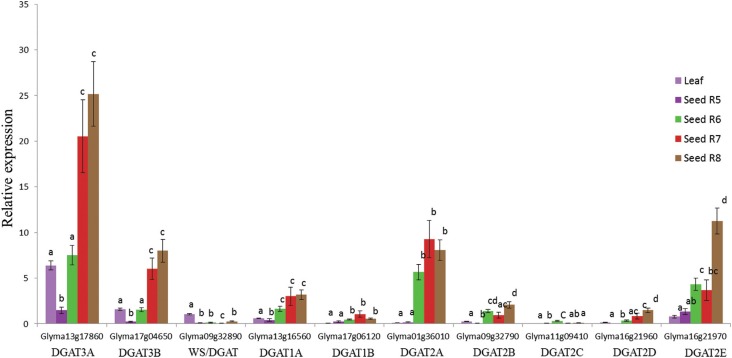
Expression profiles of the *DGAT1, DGAT2, DGAT3* and *WS*/*DGAT* genes during soybean seed development using RT-qPCR analysis. Expression profiles of each *DGAT3* (Glyma13g17860, Glyma17g04650), *WS*/*DGAT* (Glyma09g32890), *DGAT1* (Glyma13g16560, Glyma17g06120) and *DGAT2* (Glyma01g36010, Glyma09g32790, Glyma11g09410, Glyma16g21960, Glyma16g21970) genes. The comparison of expression profiles between *DGAT3* and *WS*/*DGAT* genes was done using an endogenous normalization with the leaf tissue of *WS*/*DGAT* gene. Standard error bars are based on four biological replicates. One-way ANOVA followed by the Tukey's test was used in the statistical analysis of the RT-qPCR data. mRNA input normalization was performed with four constitutive genes (*Actin, ELF, CPY* and *TUA*).

Subsequently, we checked the expression profile of putative soybean *DGAT3* and *WS/DGAT* genes, as well as the expression of *DGAT1* and *DGAT2* genes throughout four seed development stages and the leaf tissue by RT-qPCR (Figure 5). The expression analysis of *DGA1* and *DGAT2* genes was performed to compare the expression levels among the four different *DGAT* types in soybean. The expression levels of the putative *DGAT3* gene was higher in the seeds than in the leaves, with higher expression from mid to late stages of soybean seed development (R7 and R8) compared with leaf tissue and initial seed development stages. This result was similar to that found for *DGAT1* and *DGAT2* genes. In contrast, the expression levels of the putative soybean *WS/DGAT* genes were higher in leaf than in seed (Figure 5). The two putative soybean *DGAT3* genes had similar expression patterns with significantly higher expression levels observed at the full maturity stage (Figure 5). *DGAT1A* and *DGAT1B* were both highly expressed from stages R6 (Full seed) to R8 (seed maturation phase). *DGAT1B* and *DGAT1A* did not show any significant differences among the R6, R7 and R8 stages. Except for *DGAT2C*, all five *DGAT2* genes presented similar expression profiles (highly expressed at R6 to R8); the genes diverged, however, in their expression amplitude throughout soybean seed development. Comparing the expression pattern of soybean *DGAT1, DGAT2, DGAT3* and *WS/DGAT*, we found *DGAT3* as the highest expressed gene among the DGAT members. Also, one putative *DGAT3* gene (Glyma13g17860) had the highest transcript levels detected, suggesting that this gene is probably involved in TAG synthesis in seed tissue.

## Discussion

The characterization of plant *DGAT* genes is highy relevant in studies directed towards the control of oilseed storage. However, more information is still required regarding the genes of TAG synthesis in plants, since different *DGAT* genes have been identified in some plant species, but are not yet well characterized. The comprehension of the evolutionary history of these genes and the presence of so many different genes encoding *DGAT*s in plants is crucial to better understand their role in TAG biosynthesis. In the present study, taking advantage of the genome data available for several species and the large amount of current analytical methods, we identified putative *DGAT3* and *WS/DGAT* genes in several plant species and present a view about their evolution. The search for *DGAT3* and *WS/DGAT* genes in plant and algae genomes revealed that putative homologous of these genes are present in most analyzed species, suggesting that these genes could have essential function(s) in the survival of these plants, as has been shown with *DGAT1* and *DGAT2* genes. The function of the *WS/DGAT* gene was first described in *Acinetobacter calcoaceticus* (Kalscheuer and Steinbuchel, 2003) and was associated to the synthesis of both wax ester and TAG. In some bacteria, TAG formation is catalyzed by this bifunctional membrane-associated enzyme. A homologous of this gene was lately identified in *A. thaliana* (Li *et al.*, 2008). It catalyzes, predominantly, the synthesis of wax esters, but also has a DGAT activity. We identified the highly conserved condensing domain with a proposed active-site motif (^228^HHXXXDG^234^) in the N-terminal region of all putative *WS/DGAT*, suggesting that all studied plants present an ortologous of this gene. This motif was suggested to be essential for catalytic activity in the acylCoA acyltransferase reactions involved in wax ester and TAG formation (Kalscheuer and Steinbuchel, 2003, Li *et al.*, 2008). *DGAT3* function was discovered and first characterized in peanuts (*Arachis hypogaea*) (Saha *et al.*, 2006) and was recently identified in arabidopsis to be involved in active recycling of 18:2 and 18:3 fatty acids (FAs) into TAG when seed oil breakdown is blocked (Hernández *et al.*, 2012). *DGAT3* is proposed to be part of an alternative pathway for TAG synthesis (Saha *et al.*, 2006; Hernández *et al.*, 2012). This pathway occurs in the cytosol and involves the acylation of monoacylglycerol to DAG and the further acylation of DAG to TAG by the action of *DGAT3*. A soluble protein with DGAT activity has also been identified in oleaginous yeast (Rani *et al.*, 2013). In addition, another soluble acyltransferase involved in TAG syntheses (LPAAT: lysophosphatidic acid acyltransferases) has been identified in Arabidopsis (Ghosh *et al.*, 2009). Although the transmembrane domains are present in *DGAT2* and in some *WS*/*DGAT* sequences, *DGAT1* is the only gene that belongs to the superfamily of membrane-bound *O*-acyltransferases (MBOAT), which have transmembrane domains and histidine within a long hydrophobic invariant region (Hofmann, 2000). All members of the MBOAT superfamily are biochemically characterized by the transfer of organic acids, typically fatty acids, onto hydroxyl groups of membrane-embedded targets (Hofmann, 2000).

The phylogenetic analysis of putative *DGAT3, WS/DGAT, DGAT1* and *DGAT2* genes in plants revealed that they form monophyletic groups, suggesting that they probably have diverged early during plant evolution, or may have independent origins, as previously shown for *DGAT1* and *DGAT2* in eukaryotes (Turchetto-Zolet *et al.*, 2011). The independent origin hypothesis is the most likely, since *WS/DGAT* genes from plants grouped together *Acinetobacter calcoaceticus WS/DGAT*, indicating that this type of DGAT has originated before plant diversification, and the DGAT activity was maintained due the importance of TAGs in all organisms. Recently we demonstrated a distinct origin for lysophosphatidic acid acyltransferases (*LPAAT*) genes, a group of genes involved in TAG synthesis (Korbes *et al.*, 2016). Another interesting result from the phylogenetic analysis was the identification of different isoforms within each DGAT groups (DGAT1, DGAT2, DGAT3 and WS/DGAT) in some plant species. This indicates that they may have originated from gene duplication during plant evolution. This demonstrates that duplication events were important for the evolution and diversification of these genes. Gene duplication has also driven the evolution and diversification of LPAAT members during plant evolution (Korbes *et al.*, 2016). This gene encodes a soluble protein that belongs to the BAHD family (Rani *et al.*, 2010). Even though the soluble DGAT identified in the oleaginous yeast *R. glutinis* was considered a member of DGAT3 (Rani *et al.*, 2013), this sequence has an uncertain position on our phylogenetic tree and more studies including a higher number of yeast species will be necessary to clarify the phylogenetic relationship between DGATs from yeast and plant species.

Comparative analyses of exon-intron organization are very important to understand rules of gene structure and organization, protein functionality and evolutionary changes among species (Wang *et al.*, 2012). Our analysis demonstrated that the putative *DGAT3* and *WS/DGAT* genes of most analyzed species present a high degree of conservation with the well-characterized *A. thaliana DGAT3* and *WS/DGAT* genes, respectively. Nonetheless, comparison analysis of the four *DGAT* genes (*DGAT1, DGAT2, DGAT3* and *WS/DGAT*) showed that they differ in their gene (exon/intron) organization, suggesting a distinct evolutionary history for these *DGAT* genes, unveiling the diversity of *DGAT*s in plant species. We also observed that the loss/gain of introns is an evolutionary pattern for *DGAT* genes evolution. The loss/gain of introns may be caused by different processes, such as insertions of transposable elements, nucleotide substitutions or indels (Roy and Penny, 2006). We identified two *DGAT1* homologous sequences that were actually part of a single gene corresponding to *DGAT1* that was interrupted by the insertion of two transposons of the Copia family in the soybean genome (Glyma09g07510, Glyma09g07520) (data not shown). Glyma09g07510 and Glyma09g07520 lacked a DAG-binding signature motif and have probably lost their DGAT function. This suggests that in DGATs, transposable element insertions could have an important role also in tintron loss and gain.

There has been increasing evidence that DGAT enzymes play a key role in TAG biosynthesis, emphasizing the importance of understanding their roles, since TAGs are fundamental to all plant species. Distinct roles of two main DGATs (DGAT1 and DGAT2) enzymes in TAG metabolism have been demonstrated by molecular and functional characterization of these genes (Liu *et al.*, 2012). The hypothesis received supported from gene expression studies, where in some plant species, *DGAT1* and *DGAT2* were shown to have different expression profiles, acting differently in some plant species, and presenting non-redundant functions in plants (Shockey *et al.*, 2006; Chen *et al.*, 2007). Examining when and where a gene is expressed in a cell or in the whole organism can provide clues to gene function. Here, we analyzed the expression profile of putative *DGAT3* and *WS/DGAT* genes in soybean and found the same diversified pattern of transcript levels in both genes. The *in silico* and RT-qPCR analyses showed distinct expression patterns for these two *DGAT3* and *WS*/*DGAT* genes in both soybean and *A. thaliana* species. The soybean and *A. thaliana DGAT3* transcripts are more ubiquitously expressed, as they are detected in several tissues, than soybean and *A. thaliana WS*/*DGAT* transcripts, which are restricted to fewer tissues. In soybean, the transcript levels for *DGAT3* were more abundant in the final stages of seed maturation, whereas *WS/DGAT* mRNA was higher in the leaf tissue samples, indicating different gene expression and distinct regulatory mechanisms. In *A. thaliana*, Li *et al.* (2008) demonstrated that the *WS*/*DGAT* (*WSD1*) gene is transcribed in flowers, top parts of stems, and leaves.

Interestingly, when comparing the expression patterns among four *DGAT* genes in soybean, we found that the putative *DGAT3* (Glyma13g17860) sequence was the most abundant one in developing soybean seeds compared to the other *DGAT* genes. The putative *DGAT3* (Glyma17g04650) gene was also highly expressed, suggesting a possible involvement of these sequences in TAG synthesis in this species, as was demonstrated for *Arachis hypogaea* (Saha *et al.*, 2006), *A. thaliana* (Hernández *et al.*, 2012) and oleaginous yeast (Rani *et al.*, 2013). A transcriptome analysis during Arabidopsis seed development showed that the expression pattern of *DGAT1* was similar to *DGAT3* (Peng and Weselake 2011), but the expression of *DGAT3* was higher during late seed maturation.

Many studies have demonstrated differences in the expression levels between *DGAT1* and *DGAT2* genes in a number of plant species. A study comparing gene expression across seed development in four different oilseeds (*Brassica napus, Ricinus comunis, Euonimus alatus* and *Tropaeolum majus*) using transcriptome analysis showed that in *B. napus DGAT1* was more expressed than *DGAT2*, but contrasting results were observed in *R. communis*, where *DGAT1* expression is essentially absent and *DGAT2* is expressed at high levels (Troncoso-Ponce *et al.*, 2011). Another study with *Ricinus communis* showed *DGAT2* is higher expressed than *DGAT1* during seed development (Cagliari *et al.*, 2010). *DGAT2* has been associated with the accumulation of unusual TAGs in *R. comunis* and in the tung tree (Shockey *et al.*, 2006; Chen *et al.*, 2007; Burgal *et al.*, 2008). *DGAT2* transcripts are also found with relatively high abundance in olive (Alagna *et al.*, 2009) and palm (Bourgis *et al.*, 2011; Tranbarger *et al.*, 2011), which typically undergo TAG accumulation.

The phylogenetic relationship among *DGAT1, DGAT2, DGAT3* and *WS*/*DGAT* and the characteristics of exon-/ntron organization, as well as of protein sequence motifs suggest that they have evolved in an independent way in plants. It is interesting to note that although these four types of DGATs present many structural differences, the DGAT activity encoded by them has been demonstrated in several plant species. Hence, the maintenance of all these different genes encoding DGAT enzymes appears to be closely associated with the increased genomic and metabolic complexity of plants, and may be explained by the essential importance of DGAT activity in triglyceride synthesis through an evolutionarily conserved process (Ichihara *et al.*, 1988; Perry and Harwood 1993). We have demonstrated that purifying selection seems to have driven the evolution of *DGAT1* and *DGAT2* genes (Turchetto Zolet et a. 2011), suggesting a functional constraint. Thus, the observed distinct expression patterns of these genes may play a pivotal role in the development of such complex organisms, highlighting the importance of gene regulation for gene function during evolution.

In summary, the approaches used in this study allowed us to present a first general view about the presence of two *DGAT* genes (*DGAT3* and *WS*/*DGAT*) in several plant species and showed a picture about their diversity and evolution in plants. We also observed that although the *DGAT1, DGAT2, DGAT3* and *WS*/*DGAT* genes encode enzymes with a common function in TAG formation, they may have divergent expression patterns in different species and in different organs and tissues within a species. The diversity of genes encoding DGAT enzymes and their involvement in the control of TAG biosynthesis reinforces the need of functional studies of all *DGAT* genes in plants. Thereby, further comparative studies of these genes in oilseed species will be essential to identify new potential target genes for the manipulation of TAG fatty acid content through biotechnology techniques.

## Supplementary material

The following online material is available for this article:

Table S1: Species, gene name, accession numbers and protein length of DGAT sequences retrieved in this study.

Table S2: Primers used in this study.

Table S3: List of selected diacylglycerol acyltranferase (DGAT) genes used in the GENEVESTIGATOR expression analysis.

Figure S1: Soybean seed development stages (R-stages) used in this study.

Figure S2: Phylogenetic relationship between plant DGAT3 and WS/DGAT protein sequences.

Figure S3: Multiple sequence alignment of predicted amino acid sequences of DGAT3 proteins.

Figure S4: Multiple sequence alignment of predicted amino acid sequences of WS/DGAT proteins.

Figure S5: Exon-intron comparison among four *DGAT* genes (*DGAT3, DGAT2, DGAT3* and *WS*/*DGAT*) from soybean and Arabidopsis.
