# Supplementary material for: Diversity and evolution of plant diacylglycerol acyltransferase (DGATs) unveiled by phylogenetic, gene structure and expression analyses
Source: Genet Mol Biol. 2016 Oct 3;39(4):524–38. doi: 10.1590/1678-4685-GMB-2016-0024 (PMC5127155; doi:10.1590/1678-4685-GMB-2016-0024)
Supplement: Supplementary file 1 [file 1415-4757-gmb-1678-4685-GMB-2016-0024-Suppl01.pdf]

Table S1 - Species, gene name, accession numbers and protein length of DGAT sequences retrieved in this study.

**Family**	**Specie**	**Taxa terminologies**	**Gene ID**	**Database**	**Access**	**Length(aa)**
Simmondsiaceae	*Simmondsia chinensis*	Sch	WS	NCBI	AAD38041	352
Brassicaceae	*Arabidopsis thaliana*	Ath	DGAT3	Phytozome	AT1G48300	285
			WS/DGAT	Phytozome	AT1G72110	479
			WS/DGAT	Phytozome	AT2G38995	488
			WS/DGAT	Phytozome	AT3G49190	522
			WS/DGAT	Phytozome	AT3G49200	507
			WS/DGAT	Phytozome	AT3G49210	518
			WS/DGAT	Phytozome	AT5G12420	480
			WS/DGAT	Phytozome	AT5G16350	488
			WS/DGAT	Phytozome	AT5G22490	482
			WS/DGAT (WSD1)	Phytozome	AT5G37300	481
			WS/DGAT	Phytozome	AT5G53380	483
			WS/DGAT	Phytozome	AT5G53390	486
			DCR	Phytozome	AT5G23940	484
	Arabidopsis lyrata	Aly	DGAT3	Phytozome	882344	360
			WS/DGAT	Phytozome	330267	500

Family	Specie	Taxa terminologies	Gene ID	Database	Access	Length(aa)
			WS/DGAT	Phytozome	321593	474
			WS/DGAT	Phytozome	918418	488
			WS/DGAT	Phytozome	906195	503
			WS/DGAT	Phytozome	485277	521
			WS/DGAT	Phytozome	485279	519
			WS/DGAT	Phytozome	476341	425
	Brassica rapa	Bra	DGAT3	Phytozome	Bra014125	356
			WS/DGAT	Phytozome	Bra000109	491
			WS/DGAT	Phytozome	Bra000110	476
			WS/DGAT	Phytozome	Bra008008	425
			WS/DGAT	Phytozome	Bra008010	424
			WS/DGAT	Phytozome	Bra018011	538
			WS/DGAT	Phytozome	Bra018412	424
			WS/DGAT	Phytozome	Bra022653	489
			WS/DGAT	Phytozome	Bra029072	490
			WS/DGAT	Phytozome	Bra029939	468
			WS/DGAT	Phytozome	Bra029939	505
			WS/DGAT	Phytozome	Bra023022	438
			WS/DGAT	Phytozome	Bra037728	472

Family	Specie	Taxa terminologies	Gene ID	Database	Access	Length(aa)
Malvaceae	*Gossypium raimondii*	Gra	DGAT3	Phytozome	Gorai_007G116800	401
			WS/DGAT	Phytozome	Gorai.001G154900	512
			WS/DGAT	Phytozome	Gorai.001G155000	510
			WS/DGAT	Phytozome	Gorai.004G114800	482
			WS/DGAT	Phytozome	Gorai.013G132800	
			WS/DGAT	Phytozome	Gorai.013G194100	
	Theobroma cacao	Tca	DGAT3	Phytozome	Thecc1EG004941t1	401
			WS/DGAT	Phytozome	Thecc1EG015402t1	540
			WS/DGAT	Phytozome	Thecc1EG005148t1	510
Euphorbiaceae	*Ricinus communis*	Rco	DGAT3	Phytozome	29889t000177	332
			WS/DGAT	Phytozome	30024m001745	436
			WS/DGAT	Phytozome	29742m001420	506
			WS/DGAT	Phytozome	30024.m001745	436
	Manihot esculenta	Mes	DGAT3	Phytozome	cassava4_1_010675m	354
			DGAT3	Phytozome	cassava4_1_011887m	323
			WS/DGAT	Phytozome	cassava4.1_006398	489
			WS/DGAT	Phytozome	cassava4.1_029077	515
			WS/DGAT	Phytozome	cassava4.1_008553m	415
			WS/DGAT	Phytozome	cassava4.1_026440m	406

Family	Specie	Taxa terminologies	Gene ID	Database	Access	Length(aa)
Salicaceae	*Populus trichocarpa*	Ptr	DGAT3	Phytozome	Potri.010G003200	351
			WS/DGAT	Phytozome	Potri.012G014200	525
			WS/DGAT	Phytozome	Potri.019G070900	497
			WS/DGAT	Phytozome	Potri.T170200	496
			WS/DGAT	Phytozome	Potri.T076800	492
Fabaceae	*Medicago truncatula*	Mtr	DGAT3	Phytozome	Medtr4g124080	341
			WS/DGAT	Phytozome	Medtr1g008160	483
	Glycine max	Gma	DGAT3	Phytozome	Glyma13g17860	327
			DGAT3	Phytozome	Glyma17g04650	338
			WS/DGAT	Phytozome	Glyma09g32890	
	Arachis hypogaea	Ahy	DGAT3	NCBI	AAX62735	345
Solanaceae	*Solanum tuberosum*	Stu	DGAT3	Phytozome	PGSC0003DMP400008124	413
			WS/DGAT	Phytozome	PGSC0003DMP400065033	519
			WS/DGAT	Phytozome	PGSC0003DMP400069241	506
			WS/DGAT	Phytozome	PGSC0003DMP400016066	450
			WS/DGAT	Phytozome	PGSC0003DMP400000246	496
	Solanum lycopersicum	Sly	DGAT3	Phytozome	Solyc12g098850_1_1	409
			WS/DGAT	Phytozome	Solyc01g011430	506
			WS/DGAT	Phytozome	Solyc03g083380	500

Family	Specie	Taxa terminologies	Gene ID	Database	Access	Length(aa)
			WS/DGAT	Phytozome	Solyc10g009430	515
			WS/DGAT	Phytozome	Solyc01g095960	505
Ranunculaceae	*Aquilegia coerulea*	Aco	DGAT3	Phytozome	Aquca_003_00301_1	370
			WS/DGAT	Phytozome	Aquca_035_00223	499
Poaceae	*Sorghum bicolor*	Sbi	DGAT3	Phytozome	Sb09g002950	407
			WS/DGAT	Phytozome	Sb03g035790	519
			WS/DGAT	Phytozome	Sb09g027970	554
			WS/DGAT	Phytozome	Sb02g001860	491
	Oryza sativa	Osa	DGAT3	Phytozome	Loc_Os05g04620	409
			WS/DGAT	Phytozome	LOC_Os01g56360	518
			WS/DGAT	Phytozome	LOC_Os05g48260	565
			WS/DGAT	Phytozome	LOC_Os01g56360	518
	Setaria italica	Sti	DGAT3	Phytozome	Si022184m	414
			WS/DGAT	Phytozome	Si021641m	558
			WS/DGAT	Phytozome	Si001086m	511
			WS/DGAT	Phytozome	Si000956m	538
			WS/DGAT	Phytozome	Si035229m	515
			WS/DGAT	Phytozome	Si029701m	477
	Zea mays	Zma	DGAT3	Phytozome	GRMZM2G122943	411

Family	Specie	Taxa terminologies	Gene ID	Database	Access	Length(aa)
			WS/DGAT	Phytozome	GRMZM2G077375	525
	Brachypodium distachyon	Bdi	DGAT3	Phytozome	Bradi2g36890	410
			WS/DGAT	Phytozome	Bradi2g17077	537
			WS/DGAT	Phytozome	Bradi3g18630	506
			WS/DGAT	Phytozome	Bradi2g51150	503
			WS/DGAT	Phytozome	Bradi2g46640	519
			WS/DGAT	Phytozome	Bradi3g18636	478
Selaginellaceae	*Selaginella moellendorfii*	Smo	DGAT3	Phytozome	406024	229
			WS/DGAT	Phytozome	80096	512
			WS/DGAT	Phytozome	235299	452
Funariaceae	*Physcomitrella patens*	Ppa	DGAT3	Phytozome	Pp1s48149V6	461
			DGAT3	Phytozome	Pp1s65_269V6	462
			WS/DGAT	Phytozome	Pp1s279_34V6	515
Mamiellaceae	*Ostreococcus lucimarinus*	Olu	DGAT3	Phytozome		
			WS/DGAT	Phytozome	94705	365
Volvocaceae	*Volvox carteri*	Vca	DGAT3	Phytozome	103228	370
			WS/DGAT	Phytozome	-	-
	Rhodotorula glutinis	Rgl	DGAT3	NCBI	ABC41546	
	Acinetobacter sp._	Aci	WS/DGAT	NCBI	YP045555	458
