# Supplementary material for: Diversity and evolution of plant diacylglycerol acyltransferase (DGATs) unveiled by phylogenetic, gene structure and expression analyses
Source: Genet Mol Biol. 2016 Oct 3;39(4):524–38. doi: 10.1590/1678-4685-GMB-2016-0024 (PMC5127155; doi:10.1590/1678-4685-GMB-2016-0024)
Supplement: Supplementary file 2 [file 1415-4757-gmb-1678-4685-GMB-2016-0024-Suppl04.pdf]

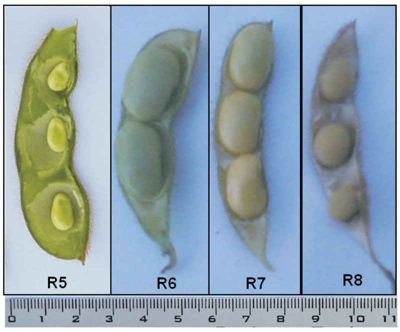

Figure S1 - Soybean seed development stages (R-stages) used in this study. R5: beginning seed; R6: full seed; R7: beginning maturity and R8: full maturity.
