# Supplementary material for: Diversity and evolution of plant diacylglycerol acyltransferase (DGATs) unveiled by phylogenetic, gene structure and expression analyses
Source: Genet Mol Biol. 2016 Oct 3;39(4):524–38. doi: 10.1590/1678-4685-GMB-2016-0024 (PMC5127155; doi:10.1590/1678-4685-GMB-2016-0024)
Supplement: Supplementary file 3 [file 1415-4757-gmb-1678-4685-GMB-2016-0024-Suppl02.pdf]

Table S2 - Nucleotide sequences of primers used in RT-qPCR analysis and amplicon length.

Acronym	Forward primer sequence (5'-3')	Reverse primer sequence (5'-3')	Amplicon size (bp)	Locus accession number
DGAT3A	CGAGAAACTTGCCATGTTCC	GTTTGGCCTTTATCTCTTGCTTT	140	Glyma13g17860
DGAT3B	GAAGGCCAAACTCAAAGCTG	CATTCGCATCCTCAACAATG	221	Glyma17g04650
WS/DGAT	GGCCACAATAGACCGAAAG	AGGCACATTGGAGAAGGCTA	287	Glyma09g32890
DGAT1A	GCGACGCCGCCAATTCG	GTTGAAGAGGCCCGCGTGACT	158	Glyma13g16560
DGAT1B	CAATTCCCAACAGCAAAACG	AAGGTTGAAGAGGCCCGC	166	Glyma17g06120
DGAT2A	GTTGACAAAAACCGAGAACCAACCA	ATTTTCATTCACCAACTCCATCCCC	223	Glyma01g36011
DGAT2B	CCTGGTTCCAGTTTTCTGCTTTGG	CAACCTCCTCAGGAGTTGGCTCGT	218	Glyma09g32790
DGAT2C	CCTGGTTCCAGTTTTCTGCTTTGG	AAACTATGGAAAGGGGCATTGGG	162	Glyma11g09410
DGAT2D	CCTGGTTCCAGTTTTCTGCTTTGG	ACCTCCTCAGGAGTTGGCTCGG	216	Glyma16g21960
DGAT2E	CTTGTTCCAGTTTTCTGCTTTG	ACCTGCTCCATTGTTGGTTCT	215	Glyma16g21970
CYP2*	CGGGACCAGTGTGCTTCTTCA	CCCCTCCACTACAAAGGCTCG	154	CF806591
ELF1B*	GTTGAAAAGCCAGGGGACA	TCTTACCCCTTGAGCGTGG	118	EV279336
ACT*	CGGTGGTTCTATCTTGGCATC	GTCTTTCGCTTCAATAACCCTA	142	BW652479
TUA*	AGGTCGGAAACTCCTGCTGG	AAGGTGTTGAAGGCGTCGTG	159	CA801144

*Primers used as reference genes
