# Supplementary material for: Diversity and evolution of plant diacylglycerol acyltransferase (DGATs) unveiled by phylogenetic, gene structure and expression analyses
Source: Genet Mol Biol. 2016 Oct 3;39(4):524–38. doi: 10.1590/1678-4685-GMB-2016-0024 (PMC5127155; doi:10.1590/1678-4685-GMB-2016-0024)
Supplement: Supplementary file 4 [file 1415-4757-gmb-1678-4685-GMB-2016-0024-Suppl05.pdf]

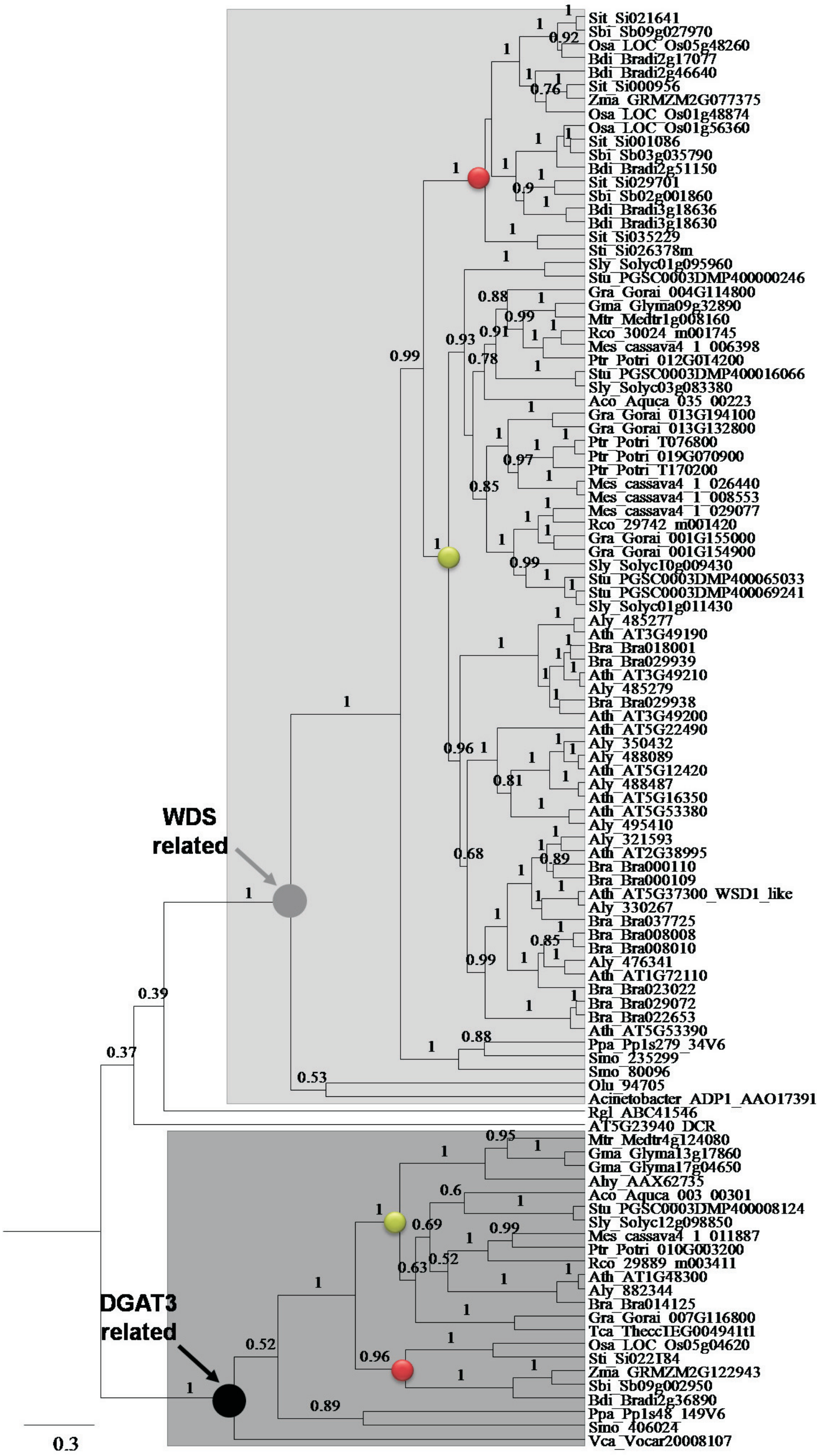

Figure S2 - Phylogenetic relationship between plant DGAT3 and WS/DGAT protein sequences. The phylogenetic analysis was performed with DGAT protein sequences from 23 species (19 plant, two algae, one fungus and one bacterium species). The species used in this analysis are listed in Table S1. Posterior probabilities are labeled above the branches; only values higher than 0.5 are presented. The red and green circles in each DGAT3 and WS/DGAT clade represent the monocot and eudicot clusters, respectively.
