# Supplementary material for: Diversity and evolution of plant diacylglycerol acyltransferase (DGATs) unveiled by phylogenetic, gene structure and expression analyses
Source: Genet Mol Biol. 2016 Oct 3;39(4):524–38. doi: 10.1590/1678-4685-GMB-2016-0024 (PMC5127155; doi:10.1590/1678-4685-GMB-2016-0024)
Supplement: Supplementary file 5 [file 1415-4757-gmb-1678-4685-GMB-2016-0024-Suppl06.pdf]

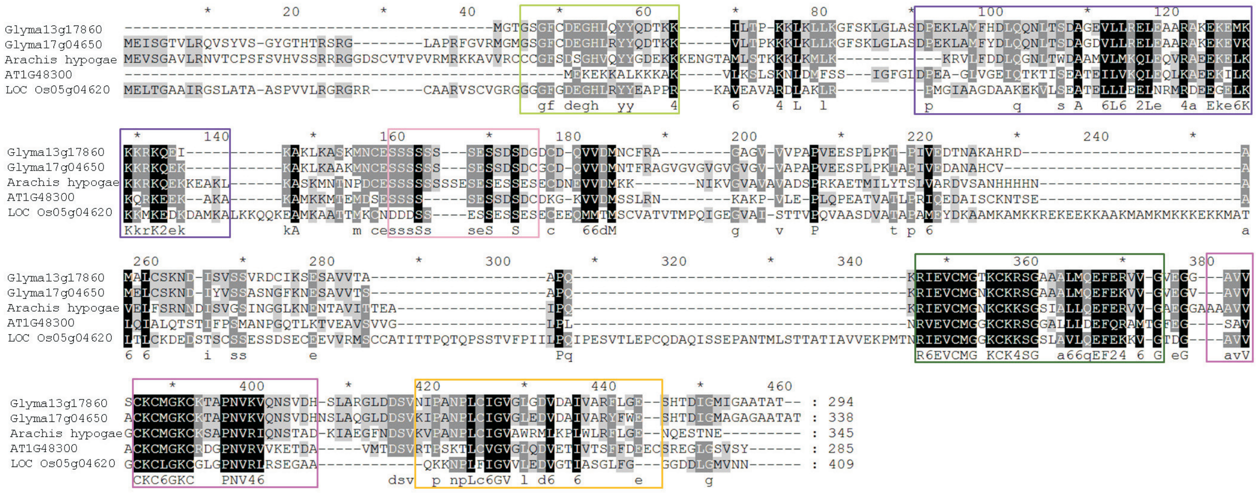

Figure S3 - Multiple sequence alignment of predicted amino acid sequences of DGAT3 proteins. The DGAT3 sequences from *G. max* (Gma), *A. thaliana* (Ath) and *O. sativa* (Osa) were aligned. Identical residues are shaded black, and similar residues are shaded gray. The color boxes indicate the conserved motifs on protein sequences shown in Figure 2B and 2C.
