# Supplementary figures and images for: Diversity and evolution of plant diacylglycerol acyltransferase (DGATs) unveiled by phylogenetic, gene structure and expression analyses
Source: Genet Mol Biol. 2016 Oct 3;39(4):524–38. doi: 10.1590/1678-4685-GMB-2016-0024 (PMC5127155; doi:10.1590/1678-4685-GMB-2016-0024)

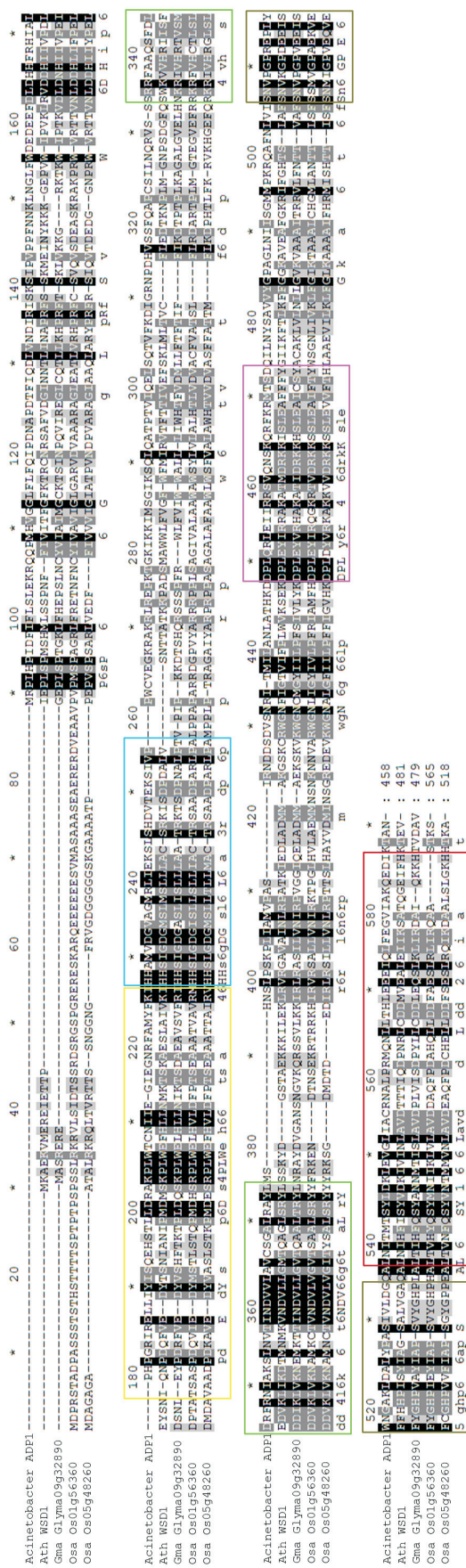

Supplement: Supplementary file 6 [file 1415-4757-gmb-1678-4685-GMB-2016-0024-Suppl07.pdf]
