# Supplementary material for: Diversity and evolution of plant diacylglycerol acyltransferase (DGATs) unveiled by phylogenetic, gene structure and expression analyses
Source: Genet Mol Biol. 2016 Oct 3;39(4):524–38. doi: 10.1590/1678-4685-GMB-2016-0024 (PMC5127155; doi:10.1590/1678-4685-GMB-2016-0024)
Supplement: Supplementary file 7 [file 1415-4757-gmb-1678-4685-GMB-2016-0024-Suppl08.pdf]

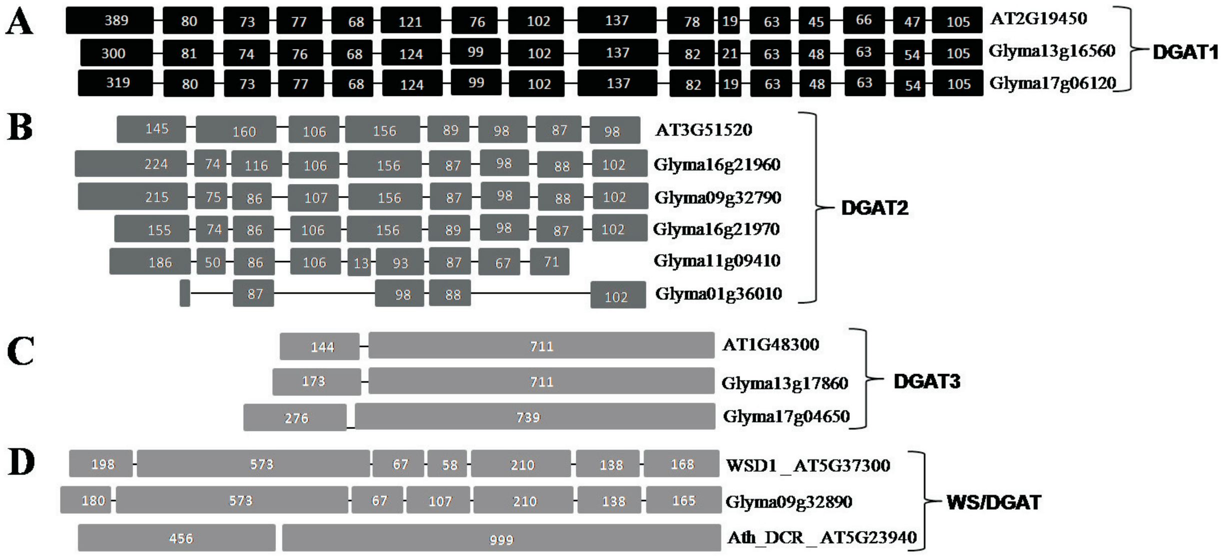

Figure S5 - Exon-intron comparison among four *DGAT* genes (*DGAT3*, *DGAT2*, *DGAT3* and *WS/DGAT*) from soybean and Arabidopsis. Exon sequences are represented by simple boxes. The size of each exon (in bp) is given. Bars represent introns. The accession number of each gene is given on the right.
