# Supplementary material for: Diversity and evolution of plant diacylglycerol acyltransferase (DGATs) unveiled by phylogenetic, gene structure and expression analyses
Source: Genet Mol Biol. 2016 Oct 3;39(4):524–38. doi: 10.1590/1678-4685-GMB-2016-0024 (PMC5127155; doi:10.1590/1678-4685-GMB-2016-0024)
Supplement: Supplementary file 8 [file 1415-4757-gmb-1678-4685-GMB-2016-0024-Suppl03.pdf]

Table S3 - Selected diacylglycerol acyltranferase (DGAT) genes used in the GENEVESTIGATOR expression analysis.

Organism	Gene symbol	Locus access	Microarray probe
Glycine max	DGAT3-related A	Glyma13g17860.1	#
	DGAT3-related B	Glyma17g04650.1	GmaAffx.63392.1.S1_at
	WS/DGAT	Glyma09g32890	Gma13644.1.S1_at
Arabidopsis thaliana	DGAT3	AT1G48300	262236_at
	WDS1	AT5G37300	249614_at
